# The Genetic Architecture of Non-Syndromic Rhegmatogenous Retinal Detachment

**DOI:** 10.3390/genes13091675

**Published:** 2022-09-19

**Authors:** Malik Moledina, David G. Charteris, Aman Chandra

**Affiliations:** 1 Department of Ophthalmology, Southend University Hospital, Mid & South Essex NHS Foundation Trust, Southend-on-Sea SS0 0RY, UK; 2 Institute of Ophthalmology, University College, London EC1V 9EL, UK; 3 Vitreoretinal Unit, Moorfields Eye Hospital NHS Foundation Trust, London EC1V 2PD, UK; 4 School of Medicine, Anglia Ruskin University, Chelmsford CM1 1SQ, UK

**Keywords:** non-syndromic retinal detachment, idiopathic retinal detachment, non-familial retinal detachment, rhegmatogenous retinal detachment

## Abstract

Rhegmatogenous retinal detachment (RRD) is the most common form of retinal detachment (RD), affecting 1 in 10,000 patients per year. The condition has significant ocular morbidity, with a sizeable proportion of patients obtaining poor visual outcomes. Despite this, the genetics underpinning Idiopathic Retinal Detachment (IRD) remain poorly understood; this is likely due to small sample sizes in relevant studies. The majority of research pertains to the well-characterised Mende lian syndromes, such as Sticklers and Wagners, associated with RRD. Nevertheless, in recent years, there has been an increasing body of literature identifying the common genetic mutations and mechanisms associated with IRD. Several recent Genomic Wide Association Studies (GWAS) studies have identified a number of genetic loci related to the development of IRD. Our review aims to provide an up-to-date summary of the significant genetic mechanisms and associations of Idiopathic RRD.

## 1. Introduction

Rhegmatogenous retinal detachment (RRD) is the most common form of retinal detachment (RD), affecting 1 in 10,000 patients per year [1]. An RRD occurs when vitreous enters the subretinal space, through a retinal break, separating the neurosensory retina from the retinal pigment epithelium [2].

It is thought that RRD occurs due to a relationship between vitreous liquefaction, adhesion, and tractional forces [2]. As individuals age, vitreous undergoes a process of liquefaction resulting in “lacunae” formation. These pockets of liquefied vitreous, subsequently come together and coalesce [3]; this process is known as synchysis. In addition, during the liquefaction process, there is an increase in optically dense structures secondary to an aggregation of collagen fibres, called syneresis [4]. Foos and Wheeler demonstrated that after a certain threshold of liquefaction, the remaining vitreous gel collapses, resulting in a Posterior Vitreous Detachment [4]; this may actualise a retinal tear which may proceed to an RRD [2,3].

The condition has significant ocular morbidity, with a sizeable proportion of patients obtaining poor visual outcomes, particularly those presenting with a fovea involving RRD [5]. Despite this, the genetics underpinning Idiopathic Retinal Detachment remain poorly understood, possibly due to small sample sizes in relevant studies. The majority of research pertains to the well-characterised Mendelian syndromes, such as Sticklers and Wagners, associated with RRD [6]. In this review, we aim to provide an understanding of the genetics of idiopathic RRD.

## 2. Complex Disease Genetics

Idiopathic Retinal Detachment is a complex genetic disease. Although there have been some notable successes utilising linkage studies in other conditions such as inflammatory bowel disease, association studies are likely to be more useful in such diseases, as they harness greater statistical power, which is useful in detecting multiple genes which individually may have a small impact [7,8]. Genomic Wide Association Studies (GWAS) have therefore become the mainstay in evaluating complex diseases, particularly since their successful advent in Age-Related Macular Degeneration and numerous subsequent examples in ophthalmic disease and endophenotype [9,10,11,12].

Typical GWAS focus on the fact that prevalent diseases may, in part, be due to common genetic variants within the population. Single nucleotide polymorphisms (SNP) are often utilised within GWAS, where high-throughput technologies genotype thousands of SNPs which are then correlated with the development of disease [9].

GWAS are structured based on a number of steps. Firstly, a large number of individuals are selected, who have the disease being investigated. A suitable comparison group is also identified; these groups then undergo DNA isolation, quality control and genotyping, after which statistical tests are performed to determine associations once quality control has been satisfied. Using the principle of linkage disequilibrium, large sections of the genome may then be identified as being associated with the phenotype or condition in question. Finally, the replication of any associations is performed [9].

## 3. Retinal Detachment Genetic Predispositions and Risk Factors

Previous studies have alluded to a strong genetic predisposition in developing idiopathic retinal detachment (IRD). Papers have estimated a cumulative lifetime risk of acquiring IRD is 2.6 times higher in relatives compared to controls (95% CI: 1.1–6.2) [13]; it has also been suggested that 8.2% of patients with IRD had a family history of RD [14]. 

Investigating 501 families in the Scottish Retinal Detachment Study, Mitry et al. estimated a siblings-recurrence risk ratio of 2.1 (95% CI 1.3–3.2) [15]. A parent-offspring recurrence risk ratio was also calculated as 2.9 (95% CI: 1.9–4.2) [15]; this study corroborated the likely strong genetic element, in developing idiopathic retinal detachment. 

Numerous risk-factors impact the development of an IRD. The most notable of these risk-factors is age; those aged 60–69, a 12.9 (95% CI: 7.7–21.7) fold increase in IRD was found, compared to individuals aged 18–29 [16]. Gender is also a key risk factor. Several recent studies have shown Males have a greater preponderance to Idiopathic Retinal Detachment, ranging from 59–68% of all cases, compared to Females [17,18]. Finally, ocular trauma is a well-documented risk factor, particularly in patients who have previously undergone phacoemulsification surgery [19]. Myopia also plays a key role, and the genetic architecture of this has been discussed elsewhere in this issue.

## 4. Genetic Mutations in Idiopathic Retinal Detachment

In investigating the genetic underpinnings of Idiopathic Retinal Detachment, the literature is relatively sparse, compared to many of the Mendelian syndromes associated with RRD. Nevertheless, the literature does describe cases of non-syndromic familial RRD inherited in an autosomal dominant manner. Richards et al. identified a novel mutation in *COL2A1*. Interestingly the family had no systemic or ocular features of Stickler’s syndrome [MIM 609508] [20]. Go et al. identified an Arg453Ter mutation in *COL2A1* in a further family with no features of syndromes associated with RD, corroborating the findings of Richards et al. [20,21].

The first GWAS study investigating the genetic architecture of Idiopathic Retinal Detachment was performed by Kirin et al. in 2013. The authors proceeded with a two-stage discovery phase where a full genome-wide scan was performed in a cohort of Scottish RD (*n* = 867) cases with ethnically matched controls (*n* = 1953); this process garnered over 300,000 SNP, of which the most promising were then tested in the second discovery phase. In the second discovery phase, the most significant SNPs were tested in populations of British and Dutch groups of cases and controls. Finally, the seven most significant SNPs from the discovery group were then utilised in the replication phase. In total 2833 cases and 7871 controls were analysed. The results of the study suggest a polygenic aetiology with multiple risk variants all producing a relatively small impact but in conjunction may account for 27.4% of the underlying RRD liability [22].

The six most significant genes identified in the study were at *TSTA3*, *LDB2* loci.54, *SS18*, *T1AM1* and *CERS2*. Of the group, only one SNP (rs267738) achieved genome-wide significance. However, in other studies, rs267738 has failed to show genome-wide significance [23]. Rs267738 is a missense coding SNP substituting Glu to Ala within the *CERS2* gene. *CESR2* encodes the protein Ceramide Synathase 2 (CerS2) which appears to be the most prevalent of the mammalian ceramide synthases and also attains the greatest tissue distribution [24]. Ceramide has been shown to be integral in caspase cascade activation leading to photoreceptor apoptosis [25,26]. Other significant genes, such as *TSTA3* and *SS18* have the ability to restructure integrins which are critical in cellular adhesion posing a potential role in the vitreoretinal interface. The products of *LBD2* and *TIAM1* are involved in cytoskeleton remodelling [22]. 

A Case-Control Study performed by Moschos and colleagues investigated associations between two specific polymorphisms on *BCL2* (rs4645878) and *BAX* (rs2279115) in 99 patients with RRD and 120 control subjects of Greek origin. The authors identified these specific polymorphisms to investigate, due to their potential role in Proliferative Vitreoretinopathy (PVR) [27,28]. Rs4645878 and rs2279115 polymorphisms have also been found to have a significant impact on the apoptotic cell death pathway. As several studies have shown, these pathways are of critical importance in photoreceptor death following a retinal detachment [27,29]; this study found the odds of IRD to be 6.89 times greater (*p* = 0.003 95% CI: 1.76–26.93 OR: 6.89) in patients with the *BAX* (rs2279115) polymorphism but found no increased susceptibility with the *BCL2* polymorphism (rs4645878) [28]. *BAX* is thought to be involved in the apoptotic pathway intracellularly. Interestingly the rs4645878 polymorphism results in decreased expression of *BAX*, though the significance of this is not fully understood in the context of IRD [30,31,32].

Quiroz-Casian and colleagues genotyped 380 Mexican patients (180 patients with IRD and 200 matched controls) for rs1042522 in the *p53* gene; this gene had previously been found to influence proliferative vitreoretinopathy development in patients of European ancestry [33]. The study found the C allele conferred 1.4 increased odds of RD (95% CI 1.01–1.9 OR:1.4) in this population. The CC homozygous genotype was also associated with an increased odds of 1.9, but failed to reach statistical significance (*p* = 0.08); this variant has been shown to impact the role of *p53* to induce apoptosis and is a recognised risk factor for malignancy [34,35]. What role this may have in the development of RD is unclear and hasn’t been replicated. Lei et al. investigated the role of *p53* in rabbit models and suggested that high levels of *p53* protected against PVR-associated RRD [36]; this was thought to be related to the role that *p53* plays in down-regulating the expression of integrins, which play a vital role in membrane contraction due to their relationship with extracellular matrix proteins [36,37].

Mutations have been identified in *ATOH7* when investigating for Non-syndromic Congenital Retinal Detachment (NSCRD) in Pakistani and Iranian cohorts. Both populations were thought to originate from consanguineous pedigrees [38,39]; this gene is thought to play a critical role in the development of retinal ganglion cells, in the absence of which, neovascular foetal vessels may infiltrate the vitreous resulting in early RD [39,40]. Despite this cohort likely to represent recessive mutations, the findings may bare some relevance to IRD.

The largest GWAS analysis to date investigating IRD was performed by Boutin et al. in 2020; it utilised data from the UK Biobank Retinal Detachment Data Set (*n* = 3977) as well as two additional datasets: The Scottish RRD study (*n* = 980) and patients recruited at London Moorfields eye hospital (*n* = 1184). Following the analysis 11 genomic-wide significant association signals were obtained at or near: *DLG5*, *TYR*, *BMP3*, *FAT3*, *LOXL1*, *ZC3H11B*, *PLCE1*, *TRIM29*, *EFEMP2*, *COL2A1* and *COL22A1*. Of these, only six loci were reproduced in the independent 23andMe dataset during the replication phase of the study (*TYR*, *ZC3H11B*, *FAT3*, *PLCE1*, *COL22A1* and *BMP3*) [23]. Despite the *LOXL1* loci not achieving replication in the 23andMe dataset, a study by Yu et al. found several *LOX* gene variants, associated with RRD in a Chinese cohort (−22G/C and 473G/A *p* < 0.001 and *p* < 0.005, respectively); it is thought the −22G/C SNP reduces the activity of *LOXL1* whilst the effect of the 473G/A polymorphism on *LOXL1* is unclear [41,42]. *LOXL1* expression has been shown in various tissue including ocular tissue [43,44,45]; its role is thought to be highly heterogenous, with overactivation and under activation leading to disease [46,47]. The role of *LOXL1* in exfoliation syndrome (a condition characterised by fibrillary white material from the lens being deposited on anterior structures of the eye) has been suggested by numerous groups [48,49]. Whilst its exact role in influencing exfoliation syndrome is unclear, it is thought that its contribution to the development of Extracellular Matrix production and maturation may play an underlying role [50,51]; this may have a similar role in IRD. 

Of the six replicated genetic loci, all have roles that could be attributed to retinal detachment aetiologies. *TYR* and *PLCE1* for example, have significant roles in retina structure and homeostasis, thus extending their impact to other ocular conditions beyond RD [23]. *FAT3* mutations are thought to produce atypical cadherins, which are calcium dependent molecules, critical in the modulation of cell behaviour [52]. Studies in mice have shown, that mutations in *FAT3*, can result in an abnormal retina formation due to the pivotal role *FAT3* Cadherins play in cell migration. Abnormal *FAT3* may adversely affect the production of a chemorepellent, preventing immature amacrine migrating toward the inner plexiform layer [23,53,54]. The resulting abnormal retinal architecture may increase the likelihood of retinal breaks [23]. A GWAS analysis performed on cases, that excluded participants with retinal breaks who did not have an RRD, resulted in the *FAT3* variant falling outside the 95% Confidence Interval; this corroborates the understanding that *FAT3* may have a greater influence on the formation of retinal breaks; a prequel to RRD [23]. 

Collagen XXII belongs to a family of proteins characterised by fibril-associated collagens with interrupted triple helices (FACIT). In studies performed in mice, this type of Collagen has been found in multiple tissues in the body including the eye. However, its function is not fully understood [55]. Collagen XXII’s role in vascular tissue and tissue structural integrity may provide insight into its role in IRD [56].

Several genes investigated for IRD have an association with Myopia which in itself is a strong independent risk factor for retinal detachment [23]. The Eye Disease Case-Control Study performed in the US, found 7.8 increased odds of IRD, in patients with myopia above −1.00 D; it also attributed 55% of non-traumatic retinal detachments, not involving prior eye surgery, to Myopia [57]; this topic is discussed in detail elsewhere in this issue. Interestingly, variants in *BMP3* (rs1960445/rs4458448) were found to cause Myopia in Caucasian populations whilst being protective in Japanese Asian populations [58]. *ZC3H11B* is expressed in the RPE as well as neural retinal tissue [59]; it was found to directly influence axial length and studies have shown its association with High Myopia [60].

A proportion of cases of Idiopathic Retinal Detachment may be clinically mischaracterised. Keser and colleagues found several of their NSCRD cases suffering from an unrecognised form of familial exudative vitreoretinopathy (FEVR) on molecular analysis. The severe phenotypes seen had previously not been associated with FEVR and the authors postulate there may be a degree of overlap between the two conditions [38]. Another study, investigating Australian pedigrees with a high incidence of RD, found many of these patients had late-onset FEVR misdiagnosed as simple RRD [61]; it is, therefore, possible that an element of IRD may be related to a variation, both in terms of genotype and phenotype, of existing conditions that perhaps have not yet been fully understood [38].

A summary of the significant genes associated with IRD, discussed in this section, has been outlined in Table 1. 

## 5. Future of Genomic Investigations

The cumulative impact of genetic factors thought to influence IRD, has been estimated at 27.4% in one study and 23% in another [22,23]. However, in line with other complex diseases, this estimated heritability is likely to explain a much smaller true phenotypic variance in the population; this gap, known as missing hereditability, is seen in a variety of complex conditions and traits [62,63]. The theories as to why this is the case are numerous. Some authors have suggested that the way in which we calculate estimated heritability is fundamentally flawed resulting in an overestimation [64]. Other explanations may relate to the study groups not being representative of the whole population. The vast majority of studies have been performed on patients with European Caucasian Ancestry which is unlikely to be representative of other ethnicities due to LD patterns and frequencies of alleles being distinct in different ethnic populations [64,65,66].

One particular hypothesis proposed is that of the “rare variant”. The rare variant hypothesis suggests a large element of inherited susceptibility to complex disease may be due to several low-frequency variants, each with moderate influence on relative risk, acting together [67]. The role of rare variants has been observed in conditions such as colorectal cancer, in which rare missense mutations, in the *APC* gene, were found to be responsible for 30–40% of non-familial colorectal cancer [68]; it has been postulated that the “rare variant” may be the largest element of the missing hereditability conundrum.

Expanding on the hypothesis-free approach, by sequencing the entire exome, may be an effective way to identify rare variants and produce novel discoveries. Whilst traditional GWAS has produced a stepwise change in our understanding of complex diseases, it is limited by its focus on common SNPs with less emphasis on rarer variants. Utilising next-generation sequencing (NGS), investigating the exome (Whole Exome sequencing (WES)), would access 85% of mutations which influence human disease [69]. Better still would be to perform Whole Genomic Sequencing (WGS). WGS has become more prevalent, predominantly in Mendelian disorders, as speeds and costs have reduced substantially. However, in complex diseases, there are still significant challenges. Both WES and WGS would require much greater sample sizes in comparison to traditional GWAS to identify putative variants [70]. Collaborations are therefore critical for investigating these conditions. In addition, the complexities around the large quantities of data garnered from WGS and WES, make the design and analysis of such studies challenging [70,71,72]. Finally, as demonstrated by numerous examples, including Boutin et al., it is possible to utilise databases that include self-reporting of diseases; this may be particularly useful in rare symptomatic acute conditions, such as IRD [23].

## 6. Conclusions

In conclusion, IRD is a complex disease influenced by polygenic aetiology. Whilst progress has been made in understanding this condition, there are still many unknowns and gaps in our understanding. With more advanced techniques such as NGS, we may be able to identify rare and intronic variants. Ultimately, this may lead to targeted preventative strategies and customised interventions which could limit the morbidity of this relatively prevalent ophthalmic emergency.

## Figures and Tables

**Table 1:** Summarises some of the significant genes associated with IRD described above.

Gene	Authors	Mechanism
*COL2A1*	Allan J Richards et al. [20], Sioe Lie Go et al. [21]	α-1 chain of type II collagen involved in tissue structural integrity.
SS18	Mirna Kirin et al. [22]	Restructuring of integrins involved in cellular adhesion possibly at the vitreoretinal interface
CERS2	Mirna Kirin et al. [22]	Ceramide Synthase 2 signalling molecules involved in Caspase Cascade Activation leading to photoreceptor apoptosis
TIAM1	Mirna Kirin et al. [22]	Cytoskeleton Remodelling
TSTA3	Mirna Kirin et al. [22]	Restructuring of integrins involved in cellular adhesion possibly at the vitreoretinal interface
LDB2	Mirna Kirin et al. [22]	Cytoskeleton Remodelling
*FAT3*	Thibaud S Boutin et al. [23]	Involved in cadherin production which is critical in the development of normal retinal architecture and cell migration
*TYR*	Thibaud S Boutin et al. [23]	Involved in retinal structure development and homeostasis.
*ZC3H11B*	Thibaud S Boutin et al. [23]	Influences axial length resulting high myopia
COL22A1	Thibaud S Boutin et al. [23]	α-1 chain of type XXII collagen involved in tissue structural integrity.
BMP3	Thibaud S Boutin et al. [23]	Associated with myopia in Caucasian populations but protective in Japanese Asian populations.
*PLCE1*	Thibaud S Boutin et al. [23]	Involved in retinal structure development and homeostasis
*LOXL1*	Thibaud S Boutin et al. [23], Honghua Yu et al. [42]	Development in Extracellular Matrix Production.
*BCL2*	Marilita M. Moschos et al. [28]	Role in the apoptotic cell death pathway of photoreceptors.
*BAX*	Marilita M. Moschos et al. [28]	Role in the apoptotic cell death pathway of photoreceptors.
*P53*	Natalia Quiroz-Casian et al. [33]	Potential role in the regulation and expression of integrins and cellular adhesion molecules.
ATOH7	Vafa Keser et al. [38], Noor M Ghiasvand et al. [39]	Development and regulation of retinal architecture in particular retinal ganglion cells

## Data Availability

The corresponding author has the right to grant, and grants on behalf of all authors, an exclusive licence on a worldwide basis to permit this article to be published.
